# Trigeminal Electrophysiology: a 2 × 2 matrix model for differential diagnosis between temporomandibular disorders and orofacial pain

**DOI:** 10.1186/1471-2474-11-141

**Published:** 2010-07-01

**Authors:** Gianni Frisardi, Giacomo Chessa, Gianfranco Sau, Flavio Frisardi

**Affiliations:** 1 "Epochè" Orofacial Pain Center, Nettuno (Rome), Italy; 2 Department of Prosthetic Rehabilitation, University of Sassari, Sassari, Italy; 3 Institute of Clinical Neurology, University of Sassari, Sassari, Italy

## Abstract

**Background:**

Pain due to temporomandibular disorders (TMDs) often has the same clinical symptoms and signs as other types of orofacial pain (OP). The possible presence of serious neurological and/or systemic organic pathologies makes differential diagnosis difficult, especially in early disease stages. In the present study, we performed a qualitative and quantitative electrophysiological evaluation of the neuromuscular responses of the trigeminal nervous system. Using the jaw jerk reflex (JJ) and the motor evoked potentials of the trigeminal roots (_b_R-MEPs) tests, we investigated the functional and organic responses of healthy subjects (control group) and patients with TMD symptoms (TMD group).

**Method:**

Thirty-three patients with temporomandibular disorder (TMD) symptoms and 36 control subjects underwent two electromyographic (EMG) tests: the jaw jerk reflex test and the motor evoked potentials of the trigeminal roots test using bilateral electrical transcranial stimulation. The mean, standard deviation, median, minimum, and maximum values were computed for the EMG absolute values. The ratio between the EMG values obtained on each side was always computed with the reference side as the numerator. For the TMD group, this side was identified as the painful side (pain side), while for the control group this was taken as the non-preferred masticatory side (non-preferred side). The 5th, 10th, 25th, 50th, 75th, 90th, and 95th percentiles were also calculated.

**Results:**

Analysis of the ratios (expressed as percentages) between the values obtained on both sides revealed a high degree of symmetry in the _b_R-MEPs ^% ^in the control (0.93 ± 0.12%) and TMD (0.91 ± 0.22%) groups. This symmetry indicated organic integrity of the trigeminal root motor fibers and correct electrode arrangement.

A degree of asymmetry of the jaw jerk's amplitude between sides (_ip_JJ%), when the mandible was kept in the intercuspal position, was found in the TMD group (0.24% ± 0.14%) with a statistically significant difference in relation to the control group (0.61% ± 0.2%). This asymmetry seemed to be primarily due to a failure to facilitate the reflex on the painful side in intercuspal position.

**Conclusions:**

In this 2 × 2 matrix diagnostic model, three different types of headache may be identified: 1) those due to organic pathologies directly and indirectly involving the trigeminal nervous system denoted as "Organic Damage"; 2) those in TMD patients; 3) other types of orofacial pain in subjects who could erroneously be considered healthy, denoted as Orofacial Pain "OP". This category of patient should be considered at risk, as organic neurological pathologies could be present and yet not directly affect the trigeminal system, at least in the early stages of the disease.

## Background

Pain due to temporomandibular disorders (TMDs) often has the same clinical symptoms and signs as other types of orofacial pain (OP). The possible presence of serious neurological and/or systemic organic pathologies makes differential diagnosis difficult, especially in early disease stages [1,2]. Frequent disagreement among researchers on appropriate diagnostic and/or therapeutic methods to be used has led to terminological, statistical, and clinical confusion, making the nosographic definition of TMDs even more complex [3]. These various diagnostic methods have a low predictive validity due to statistical errors, lack of clinical standardization, and technological limitations due to the design of diagnostic devices used [4].

In the present study, we performed a qualitative and quantitative electrophysiological evaluation of the neuromuscular responses of the trigeminal nervous system. Using the jaw jerk reflex (JJ) and the motor evoked potentials of the trigeminal roots (_b_R-MEPs) tests, we investigated the functional and organic responses of healthy subjects (control group) and patients with TMD symptoms (TMD group). We sought to construct a selective diagnostic model for TMDs and organic, especially neurological, pathologies that in the early disease stages may be confused with TMDs. Trigeminal functional responses were defined as the trigeminal reflex responses (JJ), while the trigeminal organic responses were defined as the masticatory muscle responses evoked by bilateral transcranial electrical stimulation (_b_R-MEPs) [5,6].

## Methods

The clinical study was carried out in the orofacial pain department of the University of Sassari. Sixty-nine subjects were tested in all. These included 33 patients reporting chronic unilateral pain and clinical characteristics typical of TMDs (TMD group, 37.4 ± 12.4 years) and 36 healthy subjects (control group, 30.8 ± 9 years). The clinical signs of TMD included TMJ noises (e.g. clicks and crepitation), partial or total joint blocking, jaw deviations on closure, and limited mouth opening (< 30 mm). All patients underwent spiral CT scans of TMJs to define the bony detail of the joint, while magnetic resonance imaging (MRI) was used to analyze the soft tissues where meniscal dislocation was present. Neurologic screening was performed in order to exclude patients affected by neurological disease such as that due to diabetes mellitus, neuropathic damage, and multiple sclerosis.

The control group of 36 subjects was a mixed group composed of a subgroup of 15 subjects with normal occlusion who had never in their lives complained of orofacial pain and another subgroup of 21 subjects also with normal occlusion who however complained of episodic orofacial pain in the form of hemicranias and/or tension headaches. "Normal occlusion" indicates that these subjects have physiological mandibular closure with no deviations or slipping in maximal intercuspation and had not undergone orthodontic treatment and/or prosthetic rehabilitations.

Neither subgroup, however, had a history of clinical signs or symptoms attributable to TMDs. All subjects were subjected to JJ and _b_R-MEP electrophysiological tests. The study was approved by the Human Research Ethics Committee at the Sassari Hospital (n° 871). All participants gave written informed consent.

### Jaw jerk reflex (JJ)

The JJ was elicited by placing the index finger over the middle of the patient's chin and the index finger was then tapped with a reflex hammer equipped with a piezoelectric sensor, which triggered recording of the level of EMG activity. Subjects held their mandibles in a very slightly clenched intercuspal position (_ip_JJ). Subjects were then asked to perform five maximal clenches, each lasting up to 3 s, with the mandible held in the intercuspal position to obtain the mean EMG value at maximal voluntary contraction (MVC). During these _ip_JJ tests, the subjects were guided by visual feedback to ensure that their EMG levels were maintained at ≅ 20% of the MVC. Electromyographic signals were recorded simultaneously (50 mSec time-window width, 100 μV per division, filter bandwidth 20-2 kHz) using surface electrodes on the right and left masseter muscles, with an electromyographic device (NGF-Nemus, EBNeuro, Italy). The JJ was averaged over 20 trials, and the peak-to-peak amplitude was measured.

### Bilateral R-MEPs of the trigeminal system (_b_R-MEPs)

Electrical transcranial stimulation (_e_TCS) of both trigeminal roots was performed using an electromyographic device (NGF-Nemus, EBNeuro, Italy) equipped with two electrostimulators. The stimulation electrodes were arranged on the skull as follows: the anode was placed at the vertex and the cathode electrodes were positioned 11-12 cm along the line joining the vertex to the acoustic meatus in the parietal region, on each side [7,8]. The electrical stimulus consisted of a square wave of 250 μSec duration at a voltage of ≅ 300 V and maximum current of 100 mA. The motor potentials evoked after _e_TCS of the right and left trigeminal roots were recorded on the right and left masseter muscles through two paired electrodes. The electromyographic setting was 20 mSec. time-window width, 2 mV per division and a filter bandwidth of 0.1-2 kHz.

The peak-to-peak amplitude was also analyzed. The amplitude of the electrical stimulus was maximized to recruit all the trigeminal motor fibers. The peak limit was considered to be reached when an increase in voltage yielded no changes in the amplitude of the muscular response.

### Measures

We first sought to identify the side on which the neuromuscular trigeminal reflex and evoked motor responses were reported (reference side). For the TMD group, this side was identified as the painful side (pain side), while for the control group this was taken as the non-preferred masticatory side (non-preferred side).

The following parameters were then analyzed for both groups: peak-to-peak JJ amplitude for each side when the jaw was maintained in an intercuspal position (_ip_JJ); peak-to-peak_b_R-MEPs amplitude for each side; percentage ratio between the EMG values obtained on the two sides for the JJ amplitudes in the rest (_rp_JJ^%^) and maximum intercuspation (_ip_JJ^%^) positions and between the sides for _b_R-MEPs amplitude values (_b_R-MEPs^%^).

The mean, standard deviation, median, minimum and maximum values were computed for the absolute values of the voltages.

For the ratios (Table 1), the following descriptive statistical parameters were computed: mean, standard deviation, median, variance, skewness, kurtosis, and difference between the mean values. The Mann-Whitney test for two independent samples was used to evaluate the intergroup difference between the median values. The ratio was computed by always using the value of the reference side in the numerator. The 5th, 10th, 25th, 50th, 75th, 90th, and 95th percentiles were also calculated (Table 2). Since kurtosis and skewness values for the sample under investigation showed an abnormal distribution, diagnostic cutoffs were set by using percentiles rather than confidence intervals.

**Table 1:** Baseline Characteristics of the Study Sample

	_b_R-MEPs%		_ip_JJ%	
	TMDs	Control	TMDs	Control
**Parameters**				
Mean	0.91	0.93	0.24	0.61
S.D.	0.22	0.12	0.14	0.2
Median	0.92	0.90	0.23	0.6
Variance	5-2	1.5-2	2.03-2	4.2-2
Skewness	-1.7	1.5	0.79	0.21
Kurtosis	4.7	3.13	-0.11	-1.1
Difference between means	0.02		0.37	
P-value (Mann-Whitney test)	0.63		1.3^-12^	
	NS		SS	

Percentage value of the _b_R-MEPs% and of the jaw jerk for the TMD and control groups when the mandible is kept in intercuspal position (_ip_JJ%). Since the P-value, for the _b_R-MEPs% is not less than 0.05 we cannot reject the null hypothesis while the P-value for _ip_JJ% is less than 0.05 the null hypothesis is rejected and is statistically significant (SS)

## Results

The mean amplitude of the _ip_JJ for the control group was 0.8 ± 0.5 mV on the non-preferred side and 1.3 ± 0.6 mV contralaterally, similar to those reported for healthy subjects in previous studies [9]. For the TMD group, these values were 0.4 ± 0.4 mV on the pain side and 1.5 ± 1.1 mV on the no pain side. The mean amplitude value of _b_R-MEPs for the control group was 4.4 ± 1.9 mV on the non-preferred side and 4.7 ± 2.1 mV contralaterally. For the TMD group, these mean values were 4.2 ± 2.9 mV on the pain side and 5.2 ± 2.5 mV contralaterally. For the TMD subjects, the ipsilateral JJ amplitude on the pain side was 9.5% of the _b_R-MEPs amplitude, while the percentage ratio of the no pain side values was 29%. The mean of the percentage value of the JJ amplitude compared with the R-MEP amplitude is ≅ 30% [10].

Analysis of the percentage ratios between the values obtained for the two sides (Table 1) revealed a high degree of symmetry in the _b_R-MEPs ^% ^in the control (0.93 ± 0.12%) and TMD (0.91 ± 0.22%) groups. A degree of asymmetry in the jaw jerk's (_ip_JJ%) EMG amplitude between the two sides was found in the TMD group (0.24% ± 0.14%) with a statistically significant difference (P-value = 1,3 ^-12^) in relation to the control group (0.61% ± 0.2%).

An analysis of the percentiles at different steps for the _ip_JJ^% ^and _b_R-MEPs^% ^for the control and TMD groups was performed to determine two cutoffs. The first, denoted as cutoff ^a ^and based on the _b_R-MEPs^% ^values, is necessary for an early differential diagnosis between patients with TMD symptoms and organic neurological pathologies involving the trigeminal nervous system. The second, denoted as cutoff ^b ^and based on _ip_JJ^% ^for the control and TMD groups, acts as a second filter that confirms the first filter and identifies the specific neuromuscular behavioral differences between TMD patients and those with other types of OP.

Taking a percentile of 5% for the control group, cutoff ^a ^(Table 2) falls at a _b_R-MEPs^% ^of 0.76, and is considered to be a reference value for organic disorders of the trigeminal system. Patients with values of < 0.76, which describes a between-side skewness > 24%, are considered to have organic damage and exit from the diagnostic model. Values > 0.76 pass onto the second filter (cutoff ^b^). Cutoff ^b ^at a percentile of 5% for the control group generates a between-side skewness of 0.32, which is considered to be a reference value in the differential diagnosis between TMDs and other kinds of OP. Values < 0.32, (skewness > 68%) between sides are characteristic of TMD patients and account for 75% of our sample. Table S1 in additional file 1, shows a matrix for cutoff ^a ^and cutoff ^b ^that allows rapid electrophysiological evaluation of clinical cases.

**Table 2:** Statistical Characteristics of the Study Sample

Percentiles	Control		TMDs	
	_**b**_**R-MEPs%**	_**ip**_**JJ%**	R-MEPs%	_**ip**_**JJ%**
5	0.76^*^	0.32^**^	0.21	5.1^-2^
10	0.81	0.35	0.74	0.1
25	0.85	0.44	0.85	0.13
50	0.9	0.60	0.92	0.23
75	0.96	0.81	1.06	0.32^***^
90	1.1	0.9	1.1	0.46
95	1.2	0.94	1.2	0.56

Percentiles of the ratios between the jaw jerk amplitude in the intercuspal position (_ip_JJ^%^) and the _b_R-MEPs, indicating the asymmetry value. One asterisk (*) denotes the reference value for the maximum limit of asymmetry for trigeminal responses of the _b_R-MEPs ^% ^(cutoff ^a^) for healthy subjects. Two asterisks (**) denote the maximum limit of asymmetry for the jaw jerk (cutoff^b^), corresponding to 75% of the TMD group (***).

## Discussion

### Neurophysiopathological considerations

**Figure 1:**
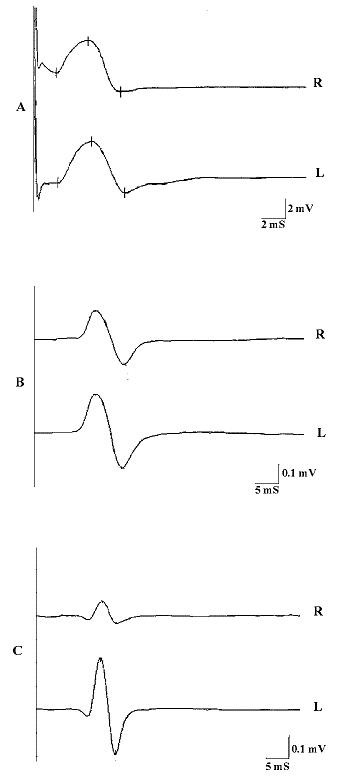
**Electrophysiological Tests**. The Figure shows: A) The neuromuscular responses evoked by transcranial electrical stimulation of the trigeminal motor roots; B) The jaw jerk reflex when the mandible was kept in rest position. Note the relative degree of symmetry between the sides; C) The jaw jerk reflex when the mandible was kept in occlusal position (intercuspal position). Note the high degree of asymmetry between the sides.

With the _e_TCS, the trigeminal fibers are depolarized along their intracranial pathways proximal to the oval foramen. The amplitude symmetry of the _b_R-MEPs^% ^in the TMD patient responses allowed us to exclude the possibility of organic damage to the motor root or technical errors in recording, including electrode positioning. (Figure 1A) Several authors [11] have reported a strong asymmetry between sides of the EMG of the temporal and the masseter muscles in TMD patients, while other authors [12] selected only TMD patients with unilateral symptoms and observed a jaw jerk asymmetry which included both latency and amplitude.

In the present study, the relative symmetry of the jaw jerk amplitude in rest position seems to be due to the centricity of the mandibular position (Figure 1B) while the asymmetry of the JJ amplitude in intercuspal position (Figure 1C) for the TMD patients seemed to be principally due to an inability to facilitate the reflex on the pain side when the jaw was maintained at maximum intercuspation. This claim stems from the fact that the mean JJ amplitude on the pain side in the TMD group corresponded to 9.5% of the ipsilateral R-MEPs amplitude, while the no-pain sides ratio was 29%.

The abnormal activity of the central drive could be explained by direct modulation of motor neurons through the cortico-bulbar system, or indirectly through modulation of the multisynaptic reflexes mediated by the lateral reticular formation. Muscular hyperactivity has been associated with psychogenic factors [13-15] and with primary pathologies of the central nervous system (CNS), as in forms of dystonia [16]. In a study in which TMD patients with unilateral symptoms and clinical signs were recruited [17], the functionalities of the cortico-bulbar and cortico-reticular systems were tested by magnetic cortical stimulation and the recovery cycle of the exteroceptive silent period of masseter muscle activity.

The electrophysiological behavior of the neuromuscular responses in these patients was identical to that of healthy subjects, thus excluding any electrophysiologically documentable form of hyperexcitability of the CNS [18]. In our study, this concept was confirmed by the fact that hypoexcitability rather than hyperexcitability was observed on the pain side.

By deviating the jaw to one side, an increase in the electromyographic activity and the JJ amplitude is induced on the mediotrusive side (contralateral to the deviation). This increase is proportional to the jaw deviation, indicating a increase in the asymmetry in the intercuspal position by means of a laterodeviation component [19]. In human experiments, voluntary contraction can activate both the skeletal-motor and fusimotor neurons [20,21]. However, it remains unclear whether the effects on the reflexes are mediated by changes in the mechanical properties of the extrafusal or intrafusal fibers or of both. Gregory et al. [22] revealed that the tendon reflex more than doubles with a voluntary muscle contraction of 5%, facilitating increases of up to 25% in voluntary contraction, after which the reflex tends to be saturated. The results of the present study seem to support this hypothesis, although no jaw deviation on the pain side has actually been documented.

Substance P (SP) and neurokinin A (NKA) are found in the synovial fluid, nerve fibers of the temporomandibular joint (TMJ), and the subnucleus caudalis of the V cranial nerve [23]. Nociceptive stimuli cause SP and NKA release, thus facilitating nociceptive reactions through a slow depolarization mediated by the tachykinin receptors, or through the release of other substances such as excitatory amino acids. The injection of mustard oil (MO) into the TMJ produces an acute inflammatory response in the tissues and a sustained increase in the excitability of the nociceptive brain-stem neurons of the subnucleus caudalis of the V cranial nerve, with activation of the jaw opening and closing reflex [24]. An experimental study [25] showed that injecting MO into mouse TMJs increases the EMG activity of the digastric and masseter muscles. This coactivation suggests muscle splinting that determines the limitation of jaw movement [26]. Furthermore, MO injection into mouse TMJs can also induce neuroplastic changes in the caudalis nociceptive neurons, reflecting a process similar to the "central sensitization" described for spinal nociceptive models [27]. Our electrophysiological tests indicated a decreased facilitatory effect of the JJ on the pain side, in contrast with the above-mentioned experimental studies pointing to an excitatory effect. However, it is not possible to exclude the different behavior of the trigeminal reflex responses at the various disease stages, for instance at the acute stage (trauma, acute meniscus dislocation, etc.) when increased EMG activity is to be expected in order to splint the TMJ.

The "vicious circle" model has also been proposed for muscle pain. Group III and IV muscle afferents should have excitatory effects on the fusimotor neurons and increase the background activity of the muscle spindles, consequently increasing their sensitivities [28]. However, fusimotors can be inhibited for 5-15 minutes after the start of pharmacologically-induced muscle inflammation, which contradicts the "vicious circle" theory [29]. Intramuscular infusion of a hypertonic saline solution has been proposed to study the excitability of spinal reflexes in man [30]. There was claimed to be an increase in the stretch reflex that could be related to the pain adaptation model [31]. Furthermore, the lack of an increase in the H-reflex suggests a peripheral rather than central effect of the neuromuscular phenomenon.

Several authors [32] have documented the functional properties of some cat brainstem neurons that received input from the neuromuscular spindles. They also investigated the effect of experimentally induced muscle pain on the central processing of the proprioceptive signals [33]. In this latter study, they identified neurons (dynamic-static and static neurons) of the subnucleus caudalis of the V cranial nerve, mainly on the medial wall of the subnucleus interpolaris adjoining the reticular formation that received indirect input from the neuromuscular spindles, probably via Probst's tract. The authors concluded that the muscle nociceptors, by acting through the interneurons, alter the fusimotor drive, which consequently modulates the sensitivity and output of the primary and secondary spindle endings. The predominant effect was exerted on the static responses of the static neurons, decreasing the mean firing rate movement induced.

Group III muscle fibers act primarily on the γ-static motor neurons, while group II muscle fibers act preferentially on γ-dynamic motor neurons [34]. Muscle pain and fatigue can inhibit the γ-static motor neurons, consequently decreasing spindle sensitivity [35]. In this neurophysiological model, a secondary central mechanism acting on proprioceptive processing is identified, and unbalanced input of the group III muscle afferents can modulate the γ-static fusimotor drive.

### Clinical considerations

During an International Association for Dental Research (IADR) Workshop in July 2008, preliminary results of the research diagnostic criteria for temporomandibular disorders RDC/TMD Validation Project were presented. Further, to come to a revised RDC/TMD, it is crucial to know not only how the test outcomes are capable of discriminating between patients with TMD pain and pain-free subjects, as studied in this Validation Project, but also, more importantly, how they discriminate between patients with TMD pain and patients with orofacial pain (OP) complaints of non-TMD origin [36]. Peripheral neuropathy, indeed, has a variety of systemic, metabolic, and toxic causes. The most common treatable causes include diabetes mellitus, hypothyroidism, and nutritional deficiencies. The diagnosis requires careful clinical assessment, judicious laboratory testing, and electrodiagnostic studies or nerve biopsy if the diagnosis remains unclear. Electrodiagnostic studies, including nerve conduction studies and electromyography can help in the differentiation of axonal versus demyelinating or mixed neuropathy [37].

In acoustic neuromas (0.3-5.0 cm), which often presents clinically with tinnitus, vertigo and hearing loss thus simulating a TMD, 73% had facial nerve impairment on electrophysiologic testing, but only 16% had facial weakness. Cranial nerve conduction was the most sensitive measurement, especially prolongation of the ipsilateral R1 latency of the blink reflex compared with that of the contralateral reflex [38].

In a study the authors presented the case of a 42-year-old woman with a 4-year history of definite multiple sclerosis (MS) and sustained contracture of the left side of the face. Needle electromyography showed continuous resting activity of irregularly firing motor unit potentials (MUP) in the left orbicularis oculi and orbicularis oris, but not in the left frontalis. Blink reflex findings were consistent with an injury in the pons, mainly in the vicinity of the left facial nucleus [39].

In our study, in conclusion, the electrophysiological behavior of the trigeminal system in the TMDs thus indicates a highly symmetrical response in the _b_R-MEPs which points an organic integrity of the motor fibers of the trigeminal roots and correct electrode arrangement. Jaw jerk was found to be comparatively symmetrical when the jaw was maintained in the rest position while it was highly asymmetric in the intercuspal position.

Extreme values, such as 5.1 ^-2 ^(Table 2) for the JJ ^% ^in the TMDs patients are compatible with a clinical condition of TMDs although they must be immediately related to the corresponding _b_R-MEPs ^% ^values.

In the 2 × 2 matrix diagnostic model (See additional file 1: Table S1 for a 2 × 2 matrix allowing rapid interpretation of the electrophysiological), three different types of headache may be identified: 1) those due to organic pathologies directly and indirectly involving the trigeminal nervous system denoted as "Organic Damage"; 2) those in TMDs patients; 3) other types of orofacial pain which respond negatively to the above-mentioned electrophysiological trigeminal tests in subjects who could erroneously be considered healthy, denoted as Orofacial Pain "OP". This category of patient should be considered at risk as organic neurological pathologies could be present and yet not directly affect the trigeminal system, at least in the early stages of the disease.

Three clinical cases of orofacial pain, the procedure for the calculation of the electrophysiological measurements and the differential reference values inserted in the 2 × 2 matrix diagnostic model for each patient are presented in this section.

The 2 × 2 matrix diagnostic model involves, for clinical simplicity, carrying out the EMG tests in order, by first performing the motor evoked potentials of the trigeminal roots (_b_R-MEPs), calculation of the amplitudes, calculation of the percentage ratios of the differences between the sides (_b_R-MEPs^%^) and insertion of the values in the diagnostic model (step 1). This first step corresponds to cutoff ^a^. The same procedure is used for the manibular reflex in the intercuspal position (_ip_JJ^%^). Insertion in the diagnostic model corresponds to cutoff ^b ^(step 2). The _b_R-MEPs and JJ were averaged over 10 trials and peak-to-peak amplitude measured.

Each time-window for EMG acquisition in the figures is divided as follows:

1. At the centre, the EMG muscle responses for the right masseter muscle (upper trace) and left masseter muscle (lower trace)

2. On the upper right hand, the markers are labeled with a number and a letter. The numbers 1 and 2 indicate the reading, the letter A indicates the onset latency, B indicates the latency and amplitude at the positive maximal peak and C the latency and amplitude at the negative maximal peak

3. At the bottom, the values for the peak-to-peak amplitudes, labeled DAmp for the right masseter muscle (1B-1C) and for the left masseter muscle (2B-2C), as well as the duration and the integral area, are shown.

### Clinical Case n°1

Male subject, aged 52 years, complaining of difficulty chewing and right TMJ pain after undergoing prosthetic dental rehabilitation 15 years earlier. Subjective episodes of paroxystic vertigo which pass rapidly, cerebellar gait, hypotrophy of the masticatory muscles, muscular trigger points, nystagmus with ocular ataxia, tongue slightly protruding on the left. Mild adiadochokinesia of the left arm, mild terminal dysmetria of the left arm, mild dysmetria on the left knee-ankle test and slight right hemifacial hypesthesia. Tendon reflexes slightly accentuated bilaterally: bicipital (C4-C5), brachioradial (C4-C5), pronator (C7-C8), tricipital (C6-C7-C8), patellar (L2-L3-L4), achilles (L5-S1-S2).

### Step 1

**Figure 2:**
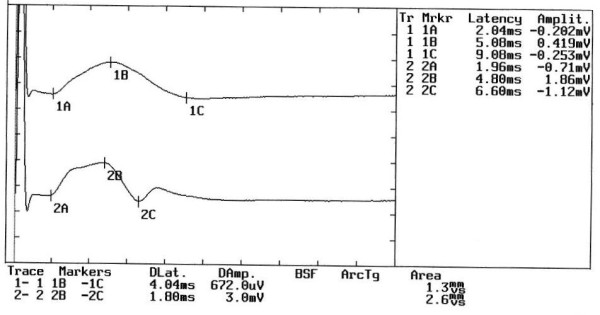
**Motor evoked potentials of the trigeminal roots in Organic Damage**. The figure shows the evident asymmetry in the amplitude of the motor evoked response by transcranial electrical stimulation of the trigeminal roots in a patient complaining of orofacial pain.

Neuromuscular responses of the _b_R-MEPs (Figure 2). The amplitude of the right masseter muscle was 0.672 mV and 3 mV on the left. The _b_R-MEPs^% ^derive from the ratio between the sides with the pain side value as the numerator. In this case the value is:(1)

The value inserted in the 2 × 2 matrix diagnostic model corresponds to a cutoff of less than 0.76 (Cutoff ^a ^< 0.76) and does not therefore allow passage to step 2.

**Figure 3:**
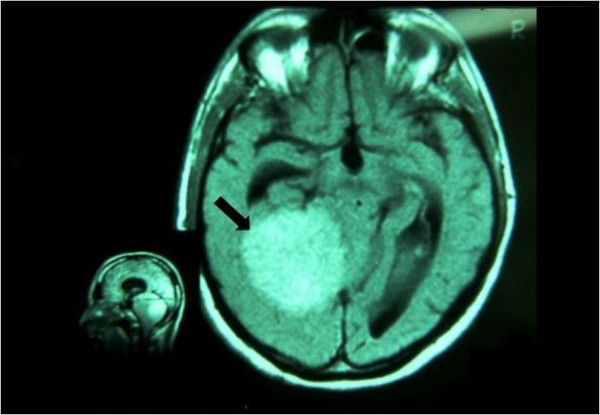
**Magnetic Resonance Imaging (MRI)**. The patient's MRI scan shows a large meningioma, 8 cm in diameter (arrow) with lateral displacement of the brainstem.

The model immediately demonstrates trigeminal organic damage and excludes the subject who is to be sent for further clinical tests. The definitive diagnosis was "Meningioma" (Figure 3).

### Clinical Case n°2

Male subject, aged 32 years, complaining of bilateral diffuse orofacial pain in the temporo-parietal region with greater intensity and frequency on the right side. The patient has been complaining since adolescence of bruxism, which was interpreted in the past as tension headaches due to bruxism and classified as a TMD.

### Step 1

**Figure 4:**
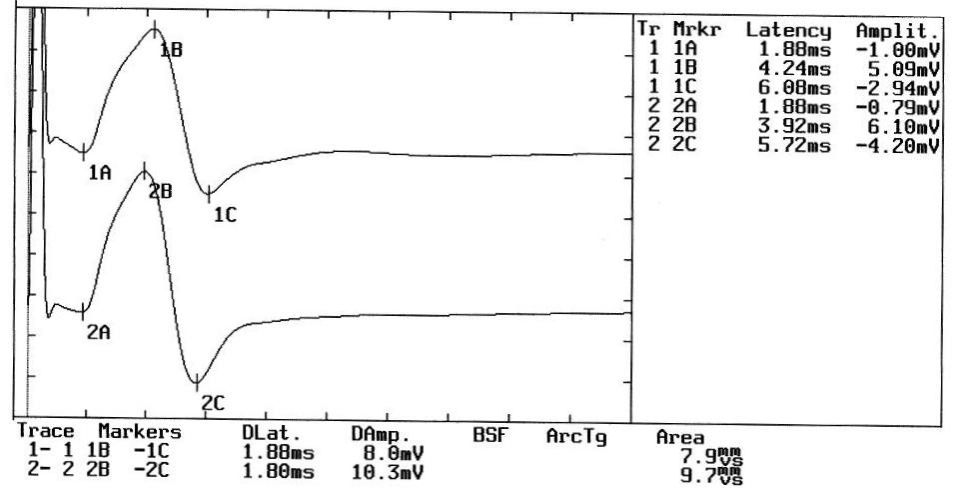
**Motor evoked potentials of the trigeminal roots in Orofacial Pain**. The figure shows the evident symmetry in the amplitude of the motor evoked response by transcranial electrical stimulation of the trigeminal roots in a patient complaining of orofacial pain erroneously interpreted as bruxism.

Neuromuscular response of the _b_R-MEPs (Figure 4). The amplitude of the right masseter muscle is 8 mV and 10.3 mV on the left. The percentage value of the _b_R-MEPs^% ^is:(2)

The value inserted in the 2 × 2 matrix diagnostic model (See additional file 1: Table S1) corresponds to a cutoff value greater than 0.76 (Cutoff ^a ^≥ 0.76) and thus allows passage to step 2.

### Step 2

**Figure 5:**
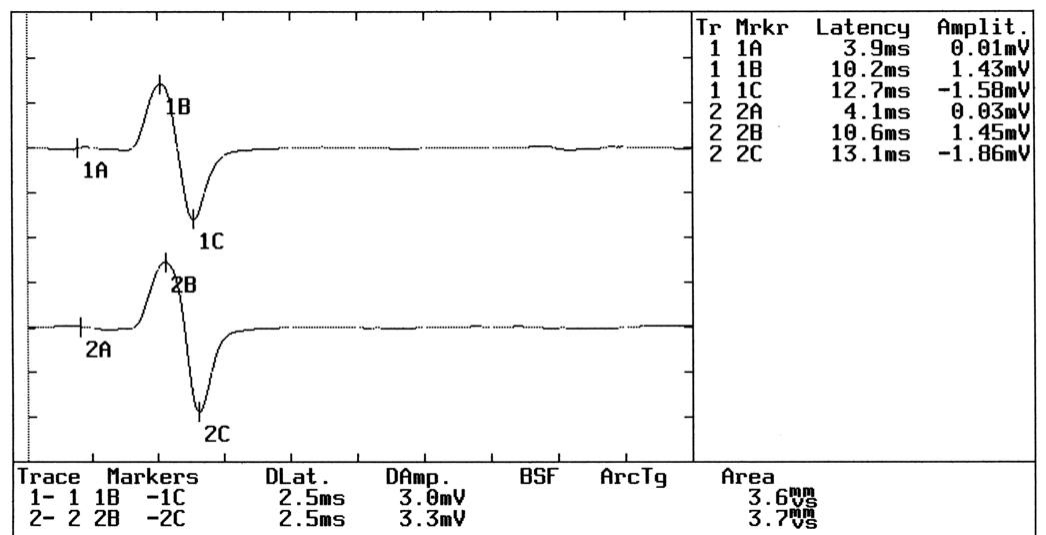
**Jaw Jerk Reflex in the intercuspal position**. The jaw jerk reflex test was performed with the mandible kept in the intercuspal position. Note the high degree of symmetry in amplitude between the two sides.

Mandibular reflex in intercuspal position (Figure 5). The amplitude of the right masseter muscle was 3 mV and 3.3 mV on the left. The _ip_JJ^% ^was:(3)

The value inserted into the 2 × 2 matrix diagnostic model (See additional file 1: Table S1) corresponds to a cutoff value greater than 0.32 (Cutoff ^b ^> 0,32) which classifies the patient as OP.

**Figure 6:**
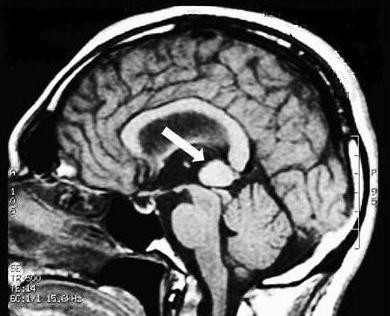
**Magnetic Resonance Imaging (MRI)**. The MR scan of the brain using contrast medium (gadolinium) showed a large pineal cavernoma.

According to this classification the subject is not considered to be suffering from TMD and is immediately excluded from the system and should undergo further clinical tests. The definitive diagnosis was "Pineal Cavernoma" (Figure 6)

### Clinical Case n°3

Female subject, aged 35 years, complaining of orofacial pain localized to the right side of the face and especially the TMJ.

The oral clinical examination showed a partial edentulism and prosthetic rehabilitation through a resin metal crown as well as signs of TMD, as crepitation and occasional blocks of the TMJ.

### Step 1

**Figure 7:**
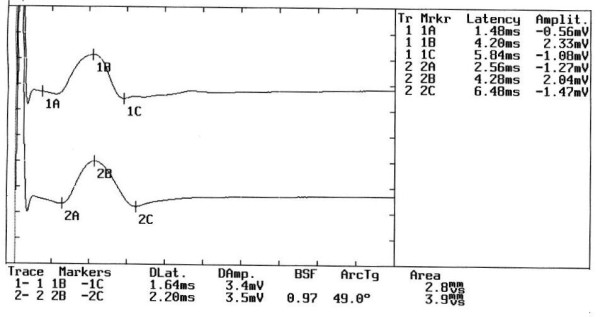
**Motor evoked potentials of the trigeminal roots in Temporomandibular Disorder**. The figure shows evident symmetry in the motor evoked response of the trigeminal roots through transcranial electrical stimulation in a patient complaining of TMDs.

Neuromuscular response of _b_R-MEPs (Figure 7). The amplitude of the right masseter muscle was 3.4 mV and 3.5 mV on the left. In this case the value is:(4)

This value inserted in the 2 × 2 matrix diagnostic model (See additional file 1: Table S1) corresponds to a cutoff value greater than 0.76 (Cutoff ^a ^≥ 0.76) and thus allows passage to step 2.

### Step 2

**Figure 8:**
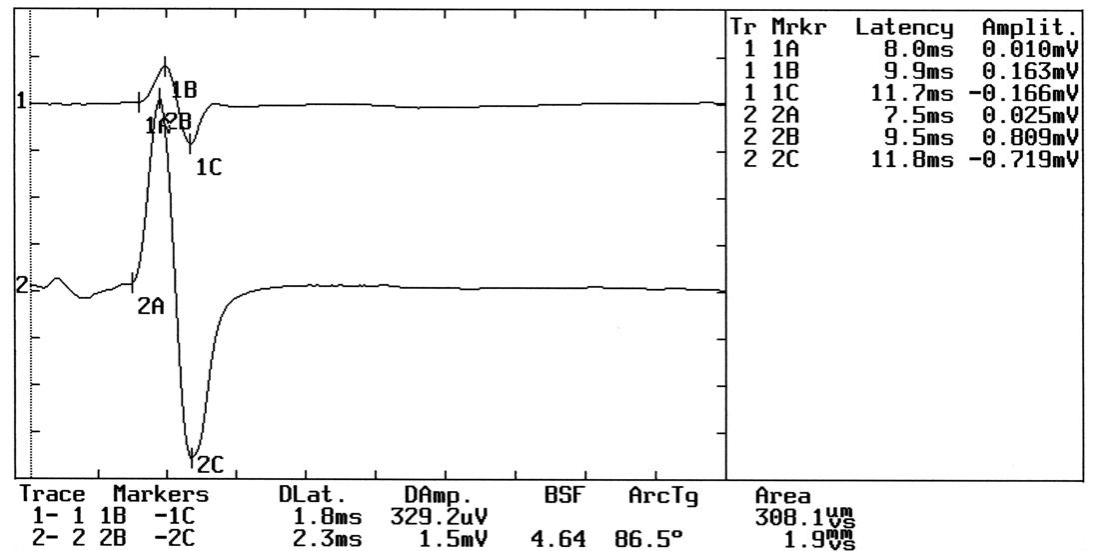
**Jaw Jerk reflex in the intercuspal position**. The figure shows the high degree of asymmetry in amplitude between the sides.

Jaw jerk reflex in intercuspal position (Figure 8). The amplitude of the right masseter muscle is 0.329 mV and 1.5 mV on the left. In this case the value is:(5)

This value inserted in the 2 × 2 matrix diagnostic model (See additional file 1: Table S1) corresponds to a limit less than 0.32 (Cutoff ^b ^< 0.32) which classifies the patient as TMD.

**Figure 9:**
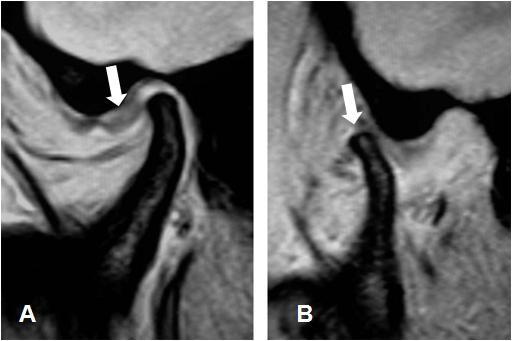
**Magnetic Resonance Imaging (MRI)**. The MR scan of the TMJ with the mouth closed and open. For details see text.

Indeed, the MRI scan shows mesial dislocation of the meniscus (Figure 9A, arrow) with the mouth closed, while with the mouth open (Figure 9B, arrow) abnormal condilar movement and reduction of the previously mesialized meniscus are observed. This clinical situation is consistent with a diagnosis of TMD caused by ligamentous laxity.

## Conclusions

This study has significantly demonstrated the behavior of the of the central and peripheral trigeminal system, i.e. the possibility of testing the organic system, using _b_R-MEPs and the functional system using the _ip_JJ. The neurophysiological findings seem to indicate a lack of facilitatory action on the painful side when the mandible was kept in intercuspal position, rather than hyperfacilitation of the central nervous trigeminal system.

Furthermore, an electrophysiological diagnostic approach was proposed in the much-discussed and controversial field of general orofacial pain. Other studies may obviously improve the model by making opportune changes.

As regards the 2 × 2 matrix diagnostic model, clearly, there may be clinical situations in which several symptoms caused by different pathologies overlap as in the concomitant presence of TMD and organic trigeminal damage. The model, in such clinical situation still has predictive validity since the first cutoff (cutoff ^a^) would immediately place the patient in the specific "Organic Damage" cell of the matrix (see Clinical Case n°1).

A patient presenting with a neurological pathology not involving the trigeminal nervous system and electrophysiologically documentable using the EMG tests presented here (see Clinical Case n°2), would obviously not be considered as healthy subject, given the presence of pain, but a patient at risk and to be referred to other departments for further tests.

The model presented therefore functions as a filter, and more precisely as a "Notch" which is specific for TMDs (see Clinical Case n°3). The model would lose some of its predictive validity in the presence of two overlapping pathologies i.e. a TMD and orofacial pain with no electrophysiologically documentable organic damage. It should be considered, however, that in these borderline clinical cases gnathological treatment in patients with TMD would rapidly eradicate their symptoms and any persisting pain would be due to underlying pathology. This complex clinical situation would require other specific electrophysiological tests such as analysis of the onset latency, the electrical and mechanical silent period of the masseter muscles, laser evoked potentials of the trigeminal system, the blink reflex, recovery cycles of the blink reflex and of the electrical masseteric silent period and, obviously, CT and MR imaging as well as laboratory blood tests.

## Competing interests

The authors declare that they have no competing interests.

## Authors' contributions

GF, GC, GFS and FF participated in the design of the study in the acquisition of data and wrote the paper. *All authors *carried out the EMG analyses, and recorded the patients' data. GF, GFS, GC and FF participated in the analysis and interpretation of data, and reviewed the manuscript. All authors read and approved the final manuscript.

## Pre-publication history

The pre-publication history for this paper can be accessed here:

http://www.biomedcentral.com/1471-2474/11/141/prepub

## Supplementary Material

Additional file 1 **A 2 × 2 matrix allowing rapid interpretation of the electrophysiological results**. In this 2 × 2 matrix diagnostic model, three different types of headache may be identified: 1) those due to organic pathologies directly and indirectly involving the trigeminal nervous system denoted as "Organic Damage"; 2) those in TMD patients; 3) other types of orofacial pain in subjects who could erroneously be considered healthy, denoted as Orofacial Pain "OP". Click here for file
