# Supplementary material for: Trigeminal Electrophysiology: a 2 × 2 matrix model for differential diagnosis between temporomandibular disorders and orofacial pain
Source: BMC Musculoskelet Disord. 2010 Jul 1;11:141. doi: 10.1186/1471-2474-11-141 (PMC2909162; doi:10.1186/1471-2474-11-141)
Supplement: Additional file 1 — A 2 × 2 matrix allowing rapid interpretation of the electrophysiological results. In this 2 × 2 matrix diagnostic model, three different types of headache may be identified: 1) those due to organic pathologies directly and indirectly involving the trigeminal nervous system denoted as "Organic Damage"; 2) those in TMD patients; 3) other types of orofacial pain in subjects who could erroneously be considered healthy, denoted as Orofacial Pain "OP". [file 1471-2474-11-141-S1.DOC]

A 2 x 2 matrix allowing rapid interpretation of the electrophysiological results

|  | **_b_Root-MEPs %**  Cutoff ^a^ < 0.76 Cutoff ^a^ ≥ 0.76 | |
| --- | --- | --- |
| **_ip_Jaw Jerk %** |  |  |
| Cutoff ^b^< 0.32 | Organic Damage | TMDs |
| Cutoff ^b^ > 0.32 |  | OP |

In this 2x2 matrix diagnostic model, three different types of headache may be identified: 1) those due to organic pathologies directly and indirectly involving the trigeminal nervous system denoted as “Organic Damage”; 2) those in TMD patients; 3) other types of orofacial pain in subjects who could erroneously be considered healthy, denoted as Orofacial Pain “OP”.
